# Yeast Tor complex 1 phosphorylates eIF4E‐binding protein, Caf20

**DOI:** 10.1111/gtc.13067

**Published:** 2023-09-12

**Authors:** Yoshiaki Kamada, Ryoko Ando, Shingo Izawa, Akira Matsuura

**Affiliations:** ^1^ National Institute for Basic Biology Okazaki Japan; ^2^ Graduate University for Advanced Studies (SOKENDAI) Hayama Kanagawa Japan; ^3^ Department of Applied Biology, Graduate School of Science and Technology Kyoto Institute of Technology Kyoto Japan; ^4^ Department of Biology, Graduate School of Science Chiba University Chiba Japan

**Keywords:** 4E‐BP, signal transduction, Tor, TORC1, translation initiation, yeast

## Abstract

Tor complex 1 (TORC1), a master regulator of cell growth, is an evolutionarily conserved protein kinase within eukaryotic organisms. To control cell growth, TORC1 governs translational processes by phosphorylating its substrate proteins in response to cellular nutritional cues. Mammalian TORC1 (mTORC1) assumes the responsibility of phosphorylating the eukaryotic translation initiation factor 4E (eIF4E)‐binding protein 1 (4E‐BP1) to regulate its interaction with eIF4E. The budding yeast *Saccharomyces cerevisiae* possesses a pair of 4E‐BP genes, *CAF20* and *EAP1*. However, the extent to which the TORC1‐4E‐BP axis regulates translational initiation in yeast remains uncertain. In this study, we demonstrated the influence of TORC1 on the phosphorylation status of Caf20 in vivo, as well as the direct phosphorylation of Caf20 by TORC1 in vitro. Furthermore, we found the TORC1‐dependent recruitment of Caf20 to the 80S ribosome. Consequently, our study proposes a plausible involvement of yeast's 4E‐BP in the efficacy of translation initiation, an aspect under the control of TORC1.

## Graphical abstract

Yeast eIF4E‐binding protein Caf20 is phosphorylated by Tor complex 1 (TORC1). Caf20 may regulate translation initiation via TORC1.

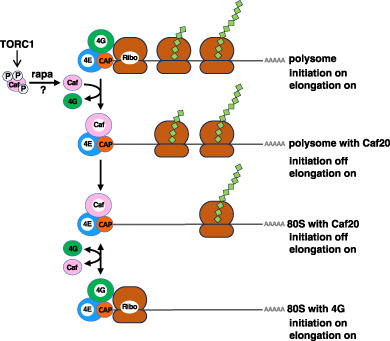

## 1 INTRODUCTION

The *T*arget *o*f *r*apamycin (Tor) is a highly conserved protein kinase found among eukaryotes (Liu & Sabatini, 2020; Loewith & Hall, 2011). Tor forms two multiprotein complexes known as Tor complex 1 (TORC1) and TORC2. TORC1, constituting a rapamycin‐sensitive branch of Tor‐signaling pathways, serves as a nutrient‐sensitive central controller of cell growth (Loewith et al., 2002). To govern cell growth, TORC1 manages the synthesis of macromolecules, particularly proteins, by phosphorylating its substrate proteins in response to cellular nutrients, specifically cellular amino acids. Moreover, TORC1 exerts a negative regulatory effect on autophagy, a process involved in protein degradation aimed at providing amino acids under starvation conditions (Noda & Ohsumi, 1998). As such, TORC1 modulates protein synthesis and amino acid consumption, as well as protein degradation and amino acid supply, in accordance with the cellular nutrient conditions.

TORC1 primarily employs two mechanisms to regulate protein synthesis. One mechanism involves the transcriptional control of genes responsible for encoding *r*ibosome *p*roteins (RP genes), genes involved in *ri*bosome *bi*ogenesis (RiBi genes), and genes responsible for the production of *r*ibosomal *RNAs* (rRNAs). In the case of mammalian TORC1 (mTORC1), it exerts its influence on RNA polymerase I and III by phosphorylating transcription factors such as UBF, TIF‐1A, and MAF1 (Liu & Sabatini, 2020). Notably, S6 kinase 1 (S6K1), whose activity is also subject to regulation through TORC1 phosphorylation, assumes a critical role in this pathway. This transcriptional control by mTORC1 found in mammals is well conserved in the budding yeast *Saccharomyces cerevisiae*. In yeast, TORC1 regulates the activity of RNA polymerase I and III, and RNA polymerase III is regulated via Sch9, a yeast counterpart of S6K1, which phosphorylates the transcription factor Maf1 (Urban et al., 2007).

The other mechanism involves translational control mediated by *e*ukaryotic *i*nitiation *f*actors (eIFs) (Kapp & Lorsch, 2004). In mammals, a crucial factor of translational control orchestrated by mTORC1 is eIF*4E*‐*b*inding *p*roteins (4E‐BPs). These 4E‐BPs exhibit an affinity for eIF4E, an essential component of the eIF4F cap‐binding complex. The binding of eIF4E‐binding protein 1 (4E‐BP1) to eIF4E prevents the association of eIF4E with eIF4G, resulting in the suppression of translational initiation. 4E‐BP1, also named PHAS‐I, is directly phosphorylated by mTORC1 at multiple sites (Brunn et al., 1997). Phosphorylated 4E‐BP1 reduces its affinity for eIF4E, thereby facilitating the assembly of the eIF4F complex (complex of eIF4A, 4E, and 4G) through eIF4E‐eIF4G binding and enhancing translational initiation. The double knockout of the 4E‐BP1 and 4E‐BP2 genes leads to the mTORC1‐independent binding of eIF4E and eIF4G, as well as protein synthesis (Thoreen et al., 2012). This suggests that the impact of mTORC1 on translational initiation is predominantly mediated by the 4E‐BPs. In yeast, there are two genes encoding 4E‐BPs, namely *CAF20* and *EAP1* (Cosentino et al., 2000; Zanchin & McCarthy, 1995). Both genes are nonessential, and the double knockout mutant remains viable. The functions of Caf20 and Eap1 appear to differ (Blewett & Goldstrohm, 2012; Higuchi et al., 2021). For instance, *CAF20* and *EAP1* influence different gene expressions, though their function in translation is significantly overlapped (Castelli et al., 2015; Cridge et al., 2010). A few studies have explored the relationship between yeast 4E‐BPs and yeast TORC1. For example, the deletion of *EAP1* confers rapamycin‐resistant cell growth (Cosentino et al., 2000). However, it remains to be determined whether yeast 4E‐BPs are directly phosphorylated by TORC1 or their functions are regulated by TORC1. In this study, we investigated whether the TORC1‐4E‐BP axis, which governs translational initiation in mammals, is conserved in yeast. Specifically, we examined whether two yeast 4E‐BPs are substrates of TORC1.

## 2 RESULTS

### 2.1 Yeast TORC1 regulates the efficiency of translation initiation

**FIGURE 1:**
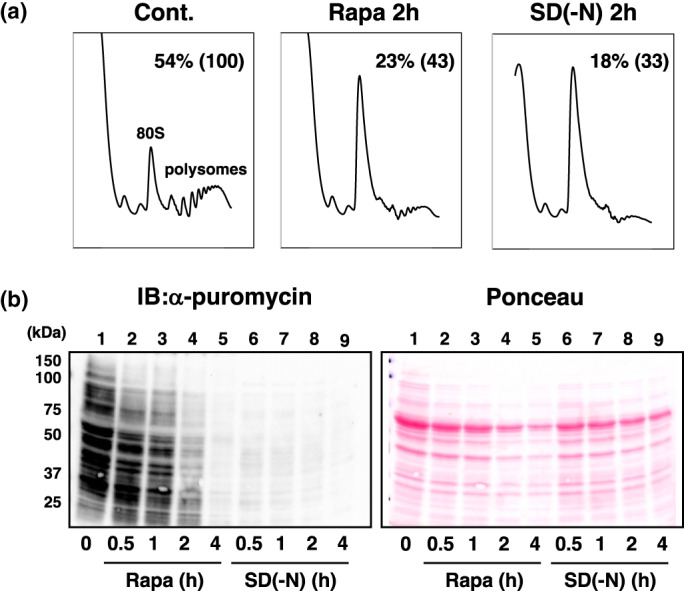
Role of TORC1 in translation. (a) Alteration of polysome profiling in response to TORC1 inhibition. Yeast cells (YYK1366) were incubated with 200 ng/mL rapamycin (Rapa) or in nitrogen starvation (SD(‐N)) for 2 h. Subsequently, the cells underwent polysome analysis. The fractions representing 80S and polysomes are indicated, along with the polysome‐to‐total ribosome ratio represented in the figure. (b) Puromycin incorporation assay. Cells (YYK1570, *pdr5*D strain) were subjected to rapamycin treatment (Lanes 2–5) or nitrogen starvation (Lanes 6–9) for the specified time period. Following this, translating polypeptides was labeled with 2 mM puromycin for 20 min. Immunoblot analysis was conducted using anti‐puromycin antibodies to analyze the protein samples.

We initiated this study by reassessing the role of TORC1 in the process of translation. We performed a thorough examination utilizing polysome profiling and puromycin incorporation assays, which allowed us to differentiate the activities of translation initiation, elongation, and protein synthesis. The polysome fraction exhibited a comparable reduction upon treatment with rapamycin (2 h) and nitrogen depletion (2 h). Conversely, the 80S fraction exhibited an increase (Figure 1a). Notably, puromycin‐labeled polypeptides were still observed in cells treated with rapamycin for up to 2 h, albeit they were nearly abolished in cells subjected to nitrogen depletion for 30 min (Figure 1b). These findings suggest that in cells treated with rapamycin for a brief period, during which TORC1 is inactivated but cellular amino acids remain accessible, the efficiency of translation initiation, which is vital for the formation of polysomes, is compromised but not completely inhibited. Consequently, protein synthesis likely takes place within 80S monosomes. Conversely, in nitrogen‐depleted cells where TORC1 is inactivated and cellular amino acids are limited, translation elongation is impeded within the monosomes. Hence, we have reaffirmed the involvement of yeast TORC1 in translation initiation, particularly with regard to its efficiency, while also acknowledging that it does not exclusively oversee translation initiation.

### 2.2 Caf20 is directly phosphorylated by TORC1

**FIGURE 2:**
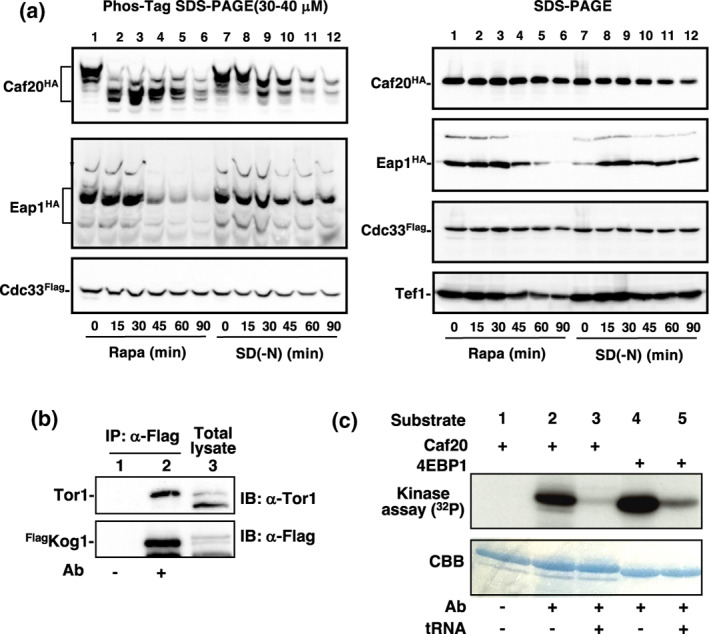
Phosphorylation of Caf20 by TORC1. (a) TORC1‐mediated phosphorylation of Caf20. Cells (YYK1228, expressing Caf20^HA^ and Cdc33^Flag^, and YYK1234, expressing Eap1^HA^ and Cdc33^Flag^) were incubated with 200 ng/mL rapamycin (Rapa, Lanes 1–6) or in nitrogen starvation (SD(‐N), Lanes 7–12) for the indicated durations. Protein samples were analyzed through immunoblotting after conducting Phos‐tag SDS‐PAGE (left) and SDS‐PAGE (right). (b) Immunoprecipitation of TORC1 from cell lysate of ^Flag^Kog1‐expressing cells (YYK1515, Lane 3) was performed with/without the presence of anti‐Flag antibody (Ab, M2). The immunoprecipitated TORC1 was subsequently detected through immunoblotting using anti‐Tor1 antibody and anti‐Flag antibody (Lane 2). (c) The immunoprecipitated TORC1 from (b) was utilized in an in vitro TORC1 kinase assay using recombinant ^His6^Caf20 (Lanes 1–3) or ^His6^4EBP1 (Lanes 4 and 5) as substrates. Transfer RNA (15 mM), an inhibitor of TORC1, was introduced into the reaction (Lanes 3 and 5).

In mammalian systems, mTORC1 plays a pivotal role in translation initiation by phosphorylating 4E‐BP1. In budding yeast, two 4E‐binding proteins, Caf20 and Eap1, have been identified. To assess whether these yeast 4E‐BPs undergo phosphorylation by TORC1, we employed Phos‐tag SDS‐PAGE and immunoblotting techniques. HA‐tagged Caf20 appeared as a slow migration band in Phos‐tag SDS‐PAGE, indicating Caf20 is phosphorylated, as previously reported (Zanchin & McCarthy, 1995). In addition, we found that the migration pattern of the HA‐tagged Caf20 band noticeably altered upon treatment with rapamycin and nitrogen depletion (15–30 min), indicating that Caf20 is phosphorylated in a TORC1‐dependent manner (Figure 2a). Conversely, the migration of the HA‐tagged Eap1 band remained unaffected by rapamycin or nitrogen depletion. Instead, the amount of Eap1 was decreased in response to rapamycin and nitrogen depletion. No discernible changes were observed in the Flag‐tagged Cdc33 (yeast eIF4E). Consequently, our focus shifted to further investigating Caf20.

Subsequently, we examined whether Caf20 serves as a substrate for TORC1 via an in vitro kinase assay. TORC1 was immunoprecipitated using Flag‐tagged Kog1, a component of TORC1, from cell lysates (Figure 2b), and TORC1 kinase activity was assessed using recombinant Caf20 and 4E‐BP1 (an artificial substrate for TORC1/TORC2 assays) (Kamada, 2017; Kamada et al., 2005). Importantly, TORC1 directly phosphorylated Caf20, along with 4E‐BP1 (Figure 2c). Previously, we reported that TORC1 activity towards 4E‐BP1 and Atg13 is inhibited by tRNA (Kamada, 2017; Otsubo et al., 2020), and we found that the phosphorylation of Caf20 is also suppressed by tRNA. These results strongly indicate that Caf20, but not Eap1, represents a novel substrate of TORC1 and is a yeast candidate counterpart to mammalian 4E‐BP1.

### 2.3 Caf20‐eIF4E complex interacts 5′ cap structure

**FIGURE 3:**
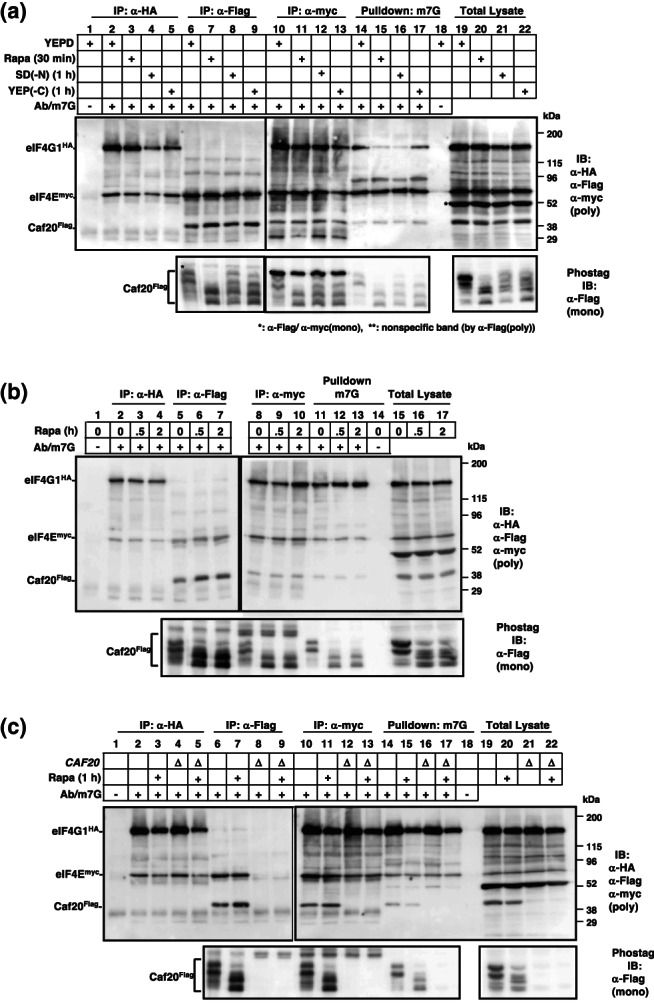
Co‐immunoprecipitation assays involving eIF4G, Caf20, and eIF4E. (a) Cells (YYK1366) expressing eIF4G1^HA^, Caf20^Flag^, and eIF4E^myc^, cultured in YEPD at 30°C, were subjected to treatment with rapamycin (Rapa) for 30 min, nitrogen starvation (SD(‐N)) for 1 h, or glucose starvation (YEP(‐C)) for 1 h. Subsequently, the cells were lysed (Lanes 19–22) and each protein was immunoprecipitated using anti‐HA (for eIF4G1^HA^ 16B12, Lanes 2–5), anti‐Flag (for Caf20^Flag^, M2, Lanes 6–9), and anti‐myc (for eIF4E^myc^, M192‐3, Lanes 10–13) monoclonal antibodies. In addition, 5′ cap‐binding proteins were pulled down using γ‐Aminophenyl‐m^7^GTP‐agarose (m7G, Lanes 14–17). Control experiments without antibody (Lane 1) or ligand (Lane 18) were also conducted. The co‐precipitated proteins were detected through immunoblotting. The protein samples were subjected to two 6.5/12.5 stepwise SDS‐PAGE gels (Lanes 1–9 and 10–22). Anti‐HA (561), anti‐Flag (PM620), and anti‐myc (562) polyclonal antibodies were used for detection (poly). Phos‐tag immunoblotting for Caf20^Flag^ using a monoclonal antibody (M2, (mono)) was also included (bottom panel). (b) Cells were treated with rapamycin for the specified duration (0, 0.5, and 2 h), and co‐immunoprecipitation assays were performed as described in (a). (c) Cells (YYK1366 and YYK1367 (*caf20*Δ)) were treated with rapamycin for 1 h, and co‐immunoprecipitation assays were carried out as described in (a).

Previous studies have reported that Caf20 exhibits an affinity for eIF4E (Cdc33) while competing with eIF4G (Tif4631/Tif4632) (Altman et al., 1997; Grüner et al., 2018; Ptushkina et al., 1998; von der Haar & McCarthy, 2002). Given that the eIF4E‐eIF4G complex plays an essential role in translation initiation, the binding of Caf20 to eIF4E, excluding eIF4G, is expected to repress or exert negative control over initiation. In mammals, the binding of 4E‐BP1 to eIF4E is regulated through the phosphorylation of 4E‐BP1 by mTORC1. Under conditions of active mTORC1, such as nutrient‐rich environments, phosphorylated 4E‐BP1 exhibits reduced affinity for eIF4E, thereby facilitating the formation of the eIF4E‐eIF4G complex. To investigate further, we examined the formation of the eIF4E‐eIF4G and eIF4E‐Caf20 complexes under varying nutrient conditions. HA‐tagged eIF4G1 (Tif4631), myc‐tagged eIF4E (Cdc33), and Flag‐tagged Caf20 were expressed in the cells, and each protein was subsequently immunoprecipitated, and the co‐precipitated proteins were detected. Consistent with previous reports (Altman et al., 1997), co‐precipitation of eIF4G1^HA^ and Caf20^Flag^ was not observed (Figure 3a, Lanes 2–5). Surprisingly, the binding of eIF4E^myc^ to eIF4G1^HA^ (Figure 3a, Lanes 2–5 and 10–13) or the binding of eIF4E^myc^ to Caf20^Flag^ (Figure 3a, Lanes 6–9 and 10–13) did not show significant changes after incubation with rapamycin, a nitrogen‐depleted medium, or a glucose‐depleted medium. Prolonged incubation with rapamycin (2 h) also had no impact on complex formation (Figure 3b). Deletion of the *CAF20* gene did not affect the status of the eIF4E^myc^‐eIF4G1^HA^ complex (Figure 3c). Furthermore, the phosphorylation status of Caf20^Flag^ did not affect the binding of eIF4E^myc^‐Caf20^Flag^ (Figure 3a–c, bottom panel). In addition, we observed that Caf20^Flag^ was pulled down by γ‐aminophenyl‐m^7^GTP‐agarose, an analog of the 7‐methylguanosine cap structure found at the 5′ ends of mRNA (Figure 3a, Lanes 14–18; Figure 3b, Lanes 11–13; Figure 3c Lanes 14 and 15). This suggests the potential formation of a ternary complex involving eIF4E‐Caf20 and mRNA. It has been proposed that eIF4F, consisting of three initiation factors (eIF4A, 4E, and 4G), binds to the 5′ end of mRNA (Kapp & Lorsch, 2004). Consequently, we further investigated the possibility of a ternary complex involving eIF4E, Caf20, and mRNA within living cells.

### 2.4 Caf20 is accumulated in the 80S monosome in response to rapamycin

**FIGURE 4:**
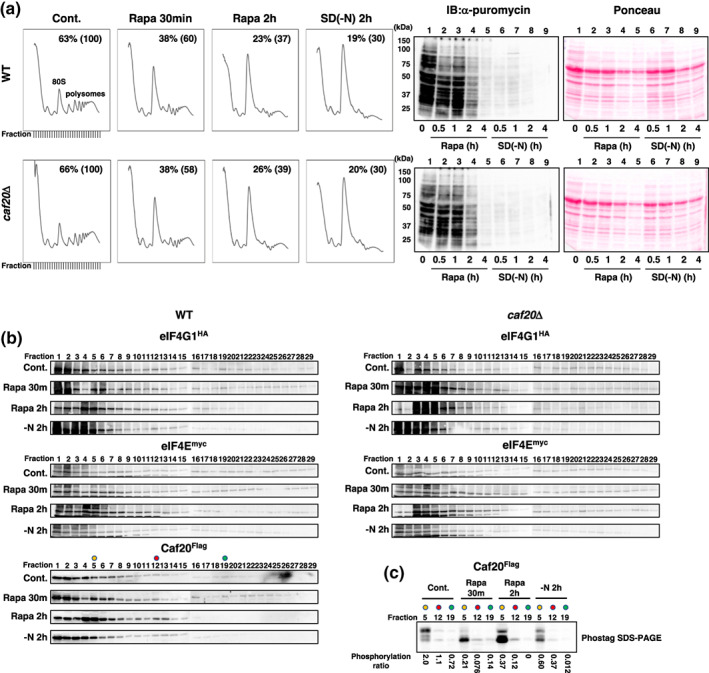
(a) (Left panels) Polysome profiling. Cells (YYK1366 and YYK1367 (*caf20*Δ)) were treated with rapamycin (30 min and 2 h) or nitrogen starvation (2 h). The fractions representing 80S and polysomes are indicated, along with the polysome‐to‐total ribosome ratio represented in the figure. Fractions collected and used for immunoblot shown (b) were indicated in the control panels. (Right panels) Puromycin incorporation assay. Cells (YYK1573 and YYK1575 (*caf20*Δ)) were analyzed as shown in Figure 1b. (b) Protein distribution. Fractions (29 samples) were collected from the polysome profile experiment (a), and eIF4G1^HA^, Caf20^Flag^, and eIF4E^myc^ were detected through immunoblotting. The fractions representing 80S and polysomes were indicated. Unedited images are shown in Figure S1. Caf20^Flag^ in Fractions #5 (yellow), #12 (red), and #19 (green) were further analyzed for phosphorylation status, as shown in (c). (c) Phos‐tag immunoblot analysis of Caf20^Flag^ present in the free fraction (Fraction #5, yellow), 80S fraction (Fraction #12, red), or polysome fraction (Fraction #19, green). The phosphorylation ratio of Caf20 is also presented.

We performed additional polysome analyses to investigate the potential inclusion of the eIF4E‐Caf20‐mRNA complex within larger structures, such as the 80S ribosome. Each fraction obtained from the polysome analysis was collected, and the presence of eIF4E^myc^, eIF4G1^HA^, and Caf20^Flag^ was detected through immunoblotting. As illustrated in Figure 4a (left panel), the 80S monosome exhibited an increase, while the polysome fraction decreased in response to rapamycin and nitrogen starvation, as described above. We also performed puromycin incorporation assays, and we did not find any apparent difference between WT and *caf20*D mutant (Figure 4a, right panel). Consequently, eIF4E^myc^ and eIF4G1^HA^ shifted from polysome fractions (Figure 4b, left panels, Lanes 16–29) to the 80S fractions (Figure 4b, left panels, Lanes 11–15) under rapamycin and nitrogen starvation conditions. For example, distribution of eIF4E^myc^ in 80S fraction (represented by Fraction #12) among monosome/polysome fraction (represented by Fraction #12 + Fraction #19) was increased from 8.7% to 64.4% by 2 h rapamycin treatment, and distribution of eIF4G1^HA^ was increased from 52.4% to 80.2%. The deletion of the *CAF20* gene did not influence the distribution of eIF4E^myc^ and eIF4G1^HA^ (Figure 4b, right panels), as evidenced by both their distribution and the results of the pull‐down assay. Caf20^Flag^ was detected in both the 80S and polysome fractions in accordance with the previous report (Castelli et al., 2015). In addition, we found that Caf20^Flag^ was notably accumulated in the 80S fractions within rapamycin‐treated cells (Figure 4b, left panels, Lanes 12–15). This observation suggests the incorporation of Caf20 into the 80S complex. We also assessed the phosphorylation status of Caf20^Flag^ in the ribosome‐free fraction (Figure 4b, Lane 5), 80S fraction (Lane 12), and polysome fractions (Lane 19). Within control cells, it is noteworthy that the phosphorylation status, denoted by the ratio of the phosphorylated form to the dephosphorylated form, of Caf20^Flag^ within the ribosome‐free, 80S, and polysome fractions stood at 2, 1.1, and 0.72, respectively (Figure 4c). This pattern of phosphorylation implies that phosphorylated Caf20 exhibits diminished affinity for ribosomes compared to its dephosphorylated counterpart.

## 3 DISCUSSION

In this investigation, we have exhibited that Caf20 is directly subjected to phosphorylation by TORC1. In addition, we found that eIF4E‐Caf20 forms a ternary assemblage with the 5′ cap structure of mRNA and that Caf20 exists within the 80S and polysome fractions. In the mammalian context, it is hypothesized that eIF4E‐bound 4E‐BP1 impedes the binding of eIF4E to mRNA due to the obligatory formation of the eIF4F complex for mRNA binding. Consequently, the regulation of translation initiation mediated by the yeast 4E‐BP appears to diverge from that of mammals. Notably, the presence of Caf20 in the 80S and polysome fraction is observed in response to rapamycin and nitrogen starvation, suggesting that the incorporation of Caf20 into the ribosome complex is of a fleeting nature. The phosphorylated state of Caf20 appears to exhibit diminished affinity towards the 80S, in contrast to its dephosphorylated counterpart. However, in this inquiry, we cannot definitively ascertain whether the recruitment of Caf20 into the ribosome complex is regulated by means of phosphorylation mediated by TORC1.

**FIGURE 5:**
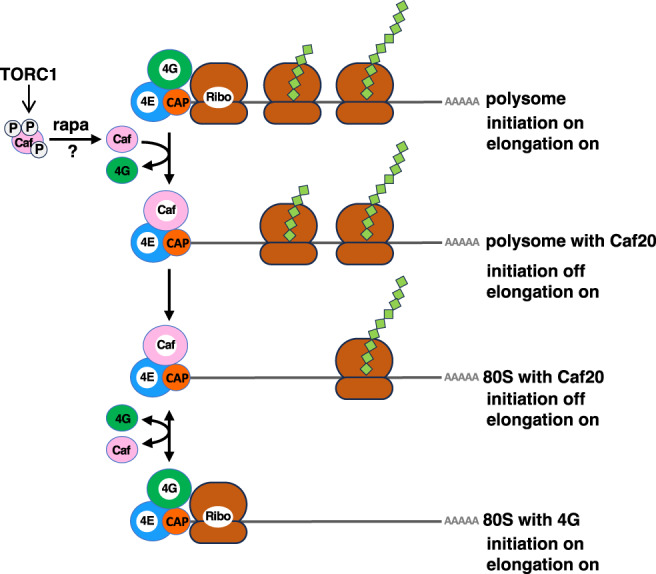
Proposed model illustrating the function of Caf20 under TORC1‐inactivated conditions. In a nutrient‐rich environment, multiple ribosomes are continuously recruited to mRNA to form polysomes. When TORC1 is inhibited by rapamycin, eIF4G is replaced by Caf20, temporarily hindering further ribosome recruitment but not affecting translational elongation. Consequently, eIF4E‐Caf20‐mRNA temporarily forms an 80S monosome with a single ribosome. Eventually, Caf20 is replaced by eIF4G, and translation initiation with ribosome recruitment resumes, allowing Caf20 to regulate translational initiation efficiency.

The subsequent topic for discussion pertains to the mechanism by which Caf20 is recruited into the 80S and polysome fractions under the TORC1 inactivated conditions. Considering that the association of eIF4E to eIF4G is indispensable for the recruitment of ribosome to the 5′ end of mRNA, it is unlikely for the eIF4E‐Caf20 complex to form the 80S/polysome without the presence of eIF4G. Instead, we propose the possibility that Caf20 may displace eIF4G from the 80S/polysome, concurrently inhibiting further ribosome recruitment (although ribosomes in the elongation phase remain unaffected). We posit that the resulting eIF4E‐Caf20‐mRNA structure is a transient event, and eIF4G is subsequently replaced to form an eIF4E‐eIF4G‐mRNA complex, thereby recruiting ribosome to resume translation as an 80S monosome. Consequently, eIF4G and Caf20 bind to eIF4E alternately to continue and cease translation initiation, respectively. Eventually, Caf20 may regulate the efficiency of ribosome recruitment, that is, the rate of translation initiation, through a cycle of exchange between eIF4G and Caf20 in the 80S under rapamycin treatment (Figure 5). Recently, it has been reported that mTORC1 adjusts translation bursting (the frequency of translational active state) by phosphorylating 4E‐BP (Livingston et al., 2023). This supports our model that transient binding of the yeast 4E‐BP modulates translational efficiency in accordance with TORC1 activity. Prolonged exposure to rapamycin progressively attenuates protein synthesis (Figure 1b), as TORC1 regulates the expression of genes involved in RiBi and RPs through Sch9 (Urban et al., 2007), ultimately resulting in a gradual reduction in ribosome supply induced by rapamycin.

Interaction of Caf20 with ribosomes and the 3′‐UTR region of mRNA in an eIF4E‐independent manner is also proposed (Castelli et al., 2015; Nwokoye et al., 2021). We generated an eIF4E‐binding motif mutant of Caf20 (Caf20‐BM), which loses affinity to eIF4E. We confirmed that this Caf20 mutant did not bind to eIF4E or m^7^GTP beads, but we found that the Caf20‐BM protein is quite unstable in rapamycin‐treated cells (Figure S2). Therefore, we could not use this mutant for further study, such as polysome profiling.

Our findings from the puromycin incorporation assay indicate that protein synthesis is not significantly impacted by short‐term rapamycin treatment (~2 h). However, these results contradict the previous study conducted by Hall and his colleagues, which reported a 90% inhibition of protein synthesis, as measured by [^35^S]‐methionine incorporation, after 2 h of rapamycin treatment (Barbet et al., 1996). This inconsistency could potentially be attributed to experimental conditions, particularly the choice of culture media. In our study, we utilized the nutrient‐rich YEPD medium for both the polysome profile and puromycin assay. In contrast, the previous study employed two different media: YEPD for the polysome profile and SD minus methionine for the [^35^S]‐methionine incorporation assay.

Is Caf20 considered a yeast 4E‐BP1? Despite the absence of discernible homology in amino acid sequences between Caf20 and 4E‐BP1, save for their shared eIF4E‐binding motif (Altman et al., 1989, 1997). Conspicuous similarities do exist between Caf20 and 4E‐BP1: both proteins bind to eIF4E, competing with eIF4G, and both are directly phosphorylated by (m)TORC1. However, the impact on translation initiation clearly differs between these two 4E‐BPs. In mammals, phosphorylation of 4E‐BP1 by mTORC1 tightly regulates its binding to eIF4E and m^7^GTP‐beads, and the double knockout of 4EBP1/2 genes leads to the continuous formation of the eIF4E‐eIF4G‐mRNA complex and confers resistance to translation inhibition by Torin 1, a Tor inhibitor (Thoreen et al., 2012). In yeast, deletion of *CAF20* does not apparently affect eIF4E‐eIF4G binding or the polysome profile. Therefore, we propose that the function of Caf20 is only partially related to that of 4E‐BP1.

## 4 EXPERIMENTAL PROCEDURES

### 4.1 Strains, plasmids, media, and genetic methods

The yeast strains, plasmids, and DNA primers utilized in this investigation are enumerated in Tables S1–S3. Conventional methodologies were employed for yeast manipulation (Kaiser et al., 1994). Primary antibodies, including HA‐epitope (16B12, COVANCE, diluted 3000‐fold and 561, MBL, diluted 3000‐fold), Flag‐epitope (M2, diluted 5000‐fold [monoclonal antibody]; Sigma and PM620, diluted 3000‐fold [polyclonal antibody]; MBL), myc‐epitope (M192‐3 [monoclonal antibody]; MBL and 562, diluted 3000‐fold [polyclonal antibody]; MBL), puromycin (3RH11, diluted 5000‐fold; Cosmo Bio), and Tor1 (sc‐11900, diluted 5000‐fold; Santa Cruz), were employed for immunoblotting at the indicated concentration in this investigation.

### 4.2 Epitope tagging of genes

C‐terminally myc‐tagged CDC33 (eIF4E), HA or Flag‐tagged CAF20, and HA‐tagged *EAP1* strains were generated as per prior studies (Longtine et al., 1998). C‐terminally HA‐tagging of TIF4631 (eIF4G1) was unsuccessful (data not shown); therefore, internally HA‐tagged TIF4631 was produced as follows: A NotI site was introduced at E375‐E377 (mutation from GAA GCT GAA to GGC GGC CGC) where evolutionary divergence was observed among *S. cerevisiae* strains, S288C, YJM1401, and Vin13. Subsequently, a NotI‐cassette containing a 3xHA fragment (Kamada et al., 1995) was inserted into this region to generate the internally HA‐tagged TIF4631 plasmid. The resulting plasmid was cloned into pRS314 and 316, effectively rescuing the slow growth phenotype of the *tif3431*Δ::KanMX strain (YYK1249).

### 4.3 Protein analyses

Yeast cells were cultivated in YEPD medium at 30°C. To induce nitrogen starvation, cells were harvested, washed thrice with distilled water, and subsequently transferred to SD(‐N) medium (0.17% yeast nitrogen base without ammonium sulfate and amino acids [Difco], 2% glucose) for incubation at 30°C. As for the puromycin incorporation assay, *pdr5*D yeast strain (YYK1570) was used because deletion of *PDR5* allows puromycin incorporation. The cells were incubated with 2 mM puromycin (Wako Chem) at room temperature for 20 min. Subsequently, cells (5 OD_600_ units) were collected and fixed with 50 μL of ice‐cold alkaline solution (0.2 N NaOH and 0.5% β‐mercaptoethanol). After 5 min of incubation on ice, 5 μL of 1.8 M NaOAc pH 5.2 and 500 mL of ice‐cold acetone were added to the sample, followed by incubation at −20°C to precipitate the protein. The protein samples were then precipitated with a microfuge for 5 min, air‐dried, and resuspended with 50 μL of SDS‐PAGE sample buffer, subsequently undergoing incubation at 65°C for 15 min. The samples were thoroughly dissolved by sonication to enable SDS‐PAGE analysis. For the evaluation of the phosphorylation status of Caf20 and Eap1, Phos‐tag Acrylamide (Wako Chem) was added to the SDS‐PAGE gel at concentrations of 15–40 μM, as per the instructions provided by the distributor. For immunoblotting, peroxidase‐conjugated goat anti‐rabbit IgG (H + L), sheep anti‐mouse IgG (H + L) (Jackson Immuno Research), or mouse anti‐goat IgG‐HRP (Santa Cruz) was utilized as the secondary antibody (diluted 10,000‐fold). Immobilon Forte Western HRP (Merck) and Light‐Capture II (ATTO) were employed for signal detection. The image of the immunoblot was meticulously cropped, and subsequently, the band's intensity was quantified employing the ImageJ software.

### 4.4 Preparation of proteins

His_6_‐tagged Caf20 and His_6_‐tagged 4EBP1, used as TORC1 substrates, were cloned into pET32a vector, expressed in *Escherichia coli* (Rosetta2 [DE3]; Merck), and purified using NTA‐agarose (Qiagen) according to the described methodology (Kamada et al., 2010).

### 4.5 Immunoprecipitation and pull‐down assay

Yeast cells cultured overnight in YEPD medium at 30°C were collected and resuspended in Z‐buffer (50 mM Tris–HCl pH 7.5, 1 M sorbitol, 1% yeast extract, 2% polypeptone, 1% glucose) containing 0.01 mg/OD600 cells of zymolyase 100T (Nacalai Tesque) to convert them into spheroplasts, with a 30‐min incubation at 30°C. The spheroplasts were then harvested, washed once with Z‐buffer, and suspended in ice‐cold IP‐buffer (1X PBS, 2 mM MgCl_2_, 1 mM Na_3_VO_4_, 7.5 mM *p*‐nitrophenylphosphate, 1% Tween‐20), supplemented with protease inhibitors (20 μg/mL leupeptin, 20 μg/mL benzamidine, 20 μg/mL aprotinin, 10 μg/mL pepstatin A, and 1 mM PMSF), at a volume of 0.1 μL/OD600 cells. The cell suspension was gently mixed and incubated on ice for 5 min to disrupt the spheroplasts. The lysate was then centrifuged at 10,000 × *g* at 4°C for 10 min, repeated twice, and the clarified lysate was incubated with 15 μL of Dynabeads protein G (50% slurry; Invitrogen) bound with or without (control) 1 μL of the indicated antibody at 4°C for 1 h with gentle rotation. For the pull‐down assay, 30 μL of γ‐aminophenyl‐m^7^GTP (C10‐spacer)‐agarose (Jena Bioscience) was used instead of protein G beads. The beads were transferred to fresh microfuge tubes, washed three times with an IP‐buffer, and subjected to further analyses.

### 4.6 In vitro TORC1 kinase assay

TORC1 kinase activity was assessed using RI kinase assays as described (Kamada, 2017). The resulting immunocomplex was washed once and suspended in 24 μL of Tes‐buffer (25 mM Tes‐KOH pH 7.3, 100 mM KCl, 10 mM MgCl_2_) containing 2 μg of substrate (^His6^Caf20 or ^His6^4EBP1), and preincubated at 30°C for 5 min. The reaction was initiated by adding 3 μL of 2 mM [γ‐32P]ATP (222 TBq/mmol Perkin Elmer) to the mixture (final concentration: 0.2 mM, 0.2 MBq/reaction), and the reaction mixture (final volume 30 μL) was further incubated at 30°C for 10 min. The reaction was stopped by adding 15 μL of 4X SDS‐PAGE sample buffer and incubating at 65°C for 5 min. The samples (20 μL) were subjected to SDS‐PAGE (12.5%), and phosphorylated proteins were analyzed by autoradiography and BAS5000 (Fuji Film).

### 4.7 Polysome analysis

Yeast cells were treated with 0.1 mg/mL cycloheximide before harvest. Cells were collected by centrifugation and fixed by liquid nitrogen. Polysome analysis was performed as described (Inada & Aiba, 2005), employing a gradient master and fractionator (107–201 M and 152–002; BioComp Instruments). The polysome ratio was calculated as the percentage of the area under polysomal ribosome peaks relative to that under total ribosome peaks.

## CONFLICT OF INTEREST STATEMENT

The authors declare no conflict of interest.

## Supporting information


**DATA S1.** Supporting Information.

**TABLE S1.** Yeast strains used in this study.
