# Supplementary material for: Yeast Tor complex 1 phosphorylates eIF4E‐binding protein, Caf20
Source: Genes Cells. 2023 Sep 12;28(11):789–99. doi: 10.1111/gtc.13067 (PMC11447835; doi:10.1111/gtc.13067)

Fig. S1 Original image of Fig. 4B

(A) Immunoblot using polyclonal antibodies against HA (detecting eIF4G1^{HA}), myc (detecting eIF4Emyc), and Flag (detecting Caf20^{Flag}, Fig. S1(B) image was taken for Fig. 4B)

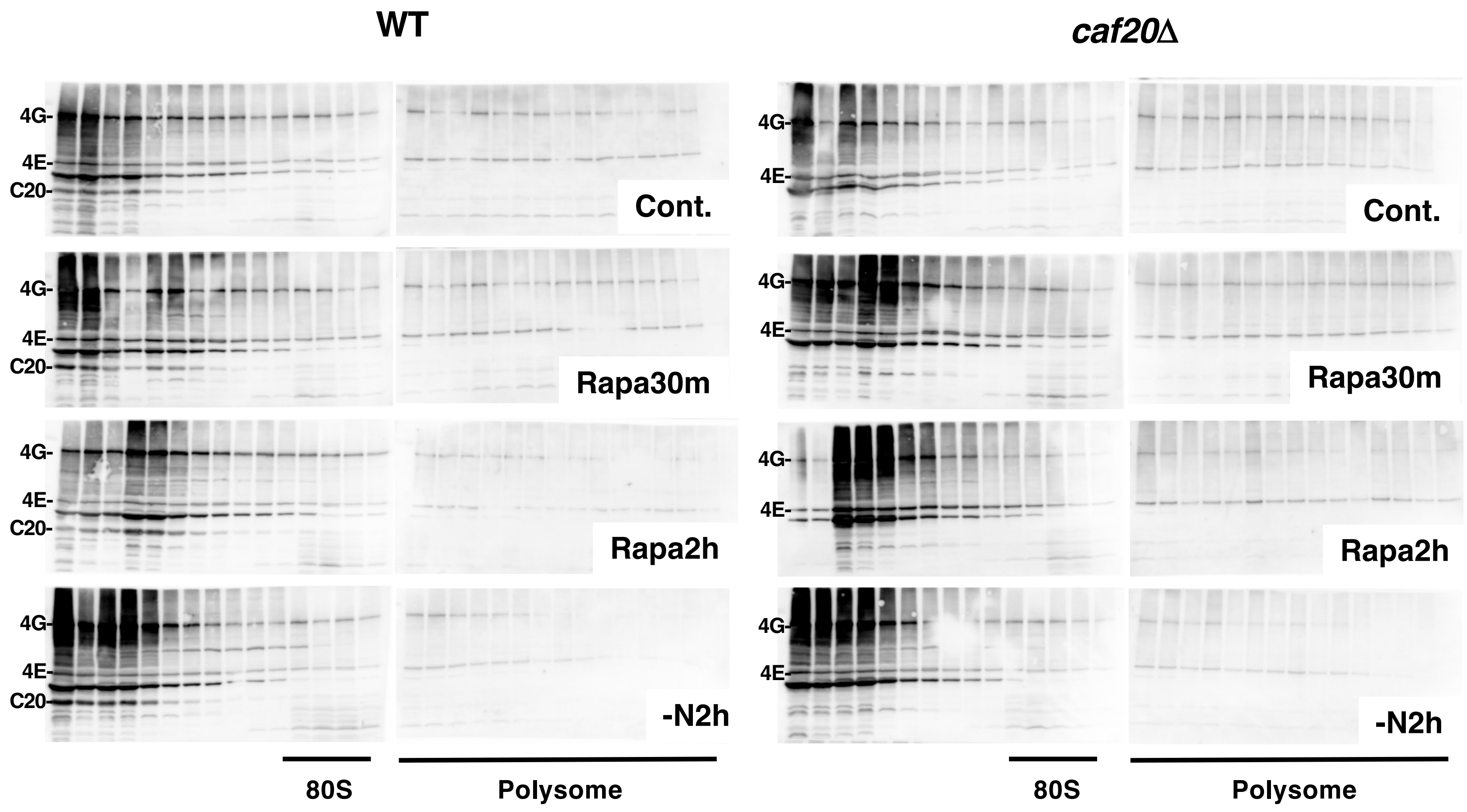

(B) Immunoblot using monoclonal antibody against Flag (detecting Caf20^{Flag}, Fig. S1(B) image was taken for Fig. 4B)

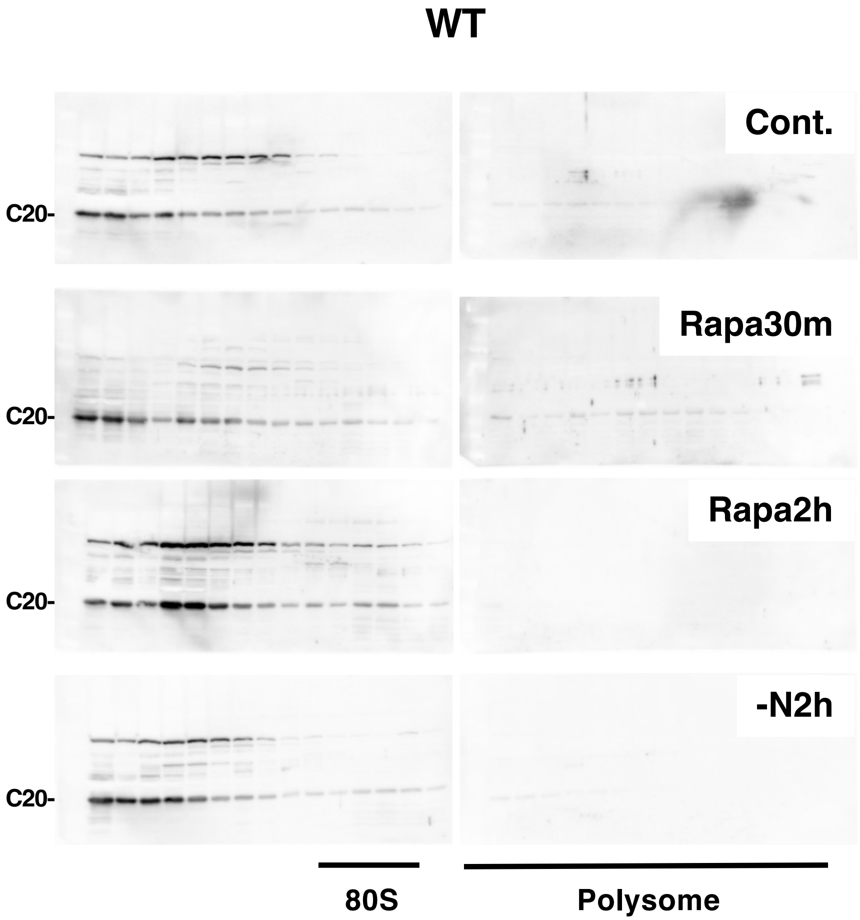

(C) Ponceau staining of membrane filter used in Fig. S1 (A) and (B)

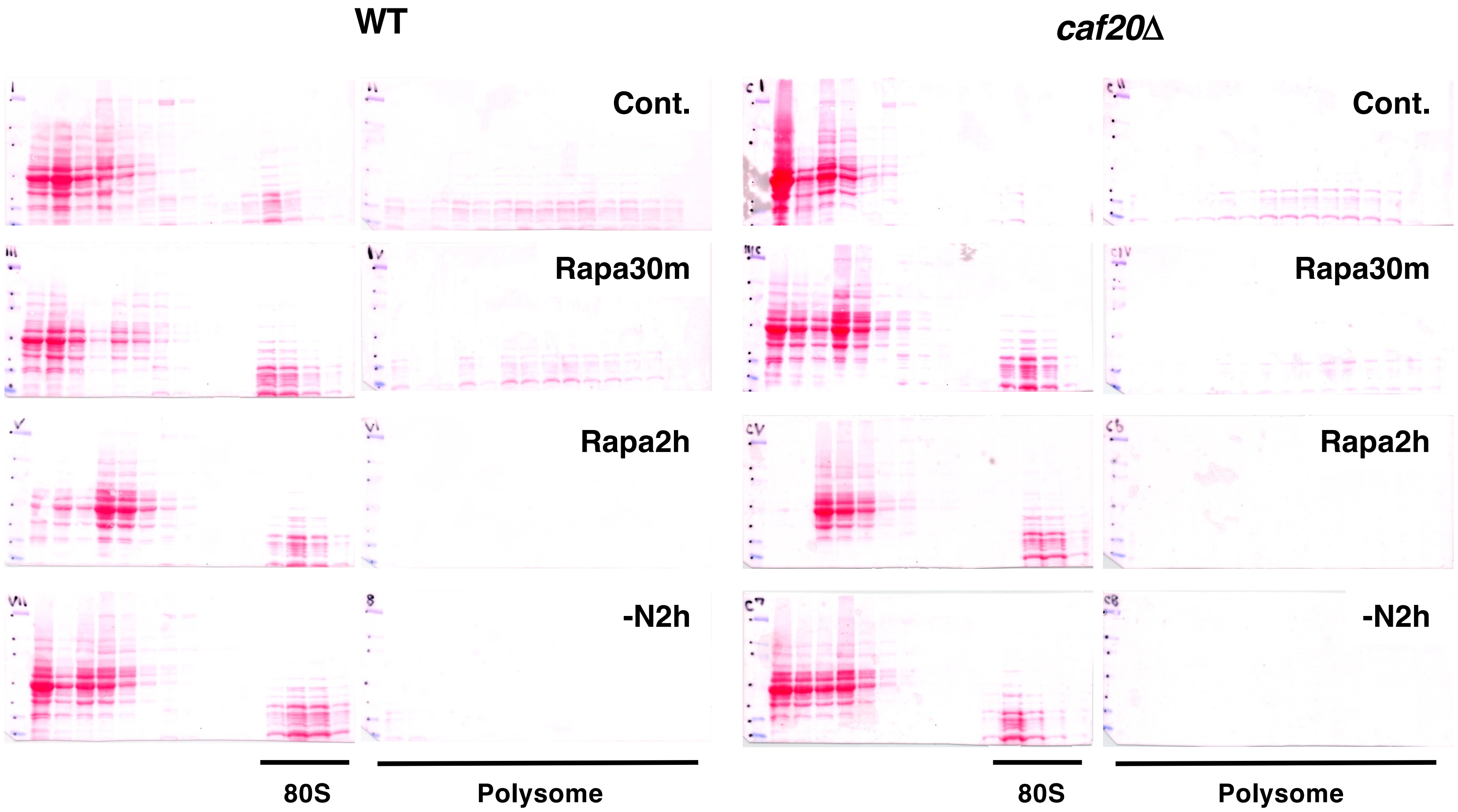

Fig. S2 Co-immunoprecipitation assay using Caf20-BM mutant
Experiment was performed as in Fig. 3 using *caf20*-BM strain.

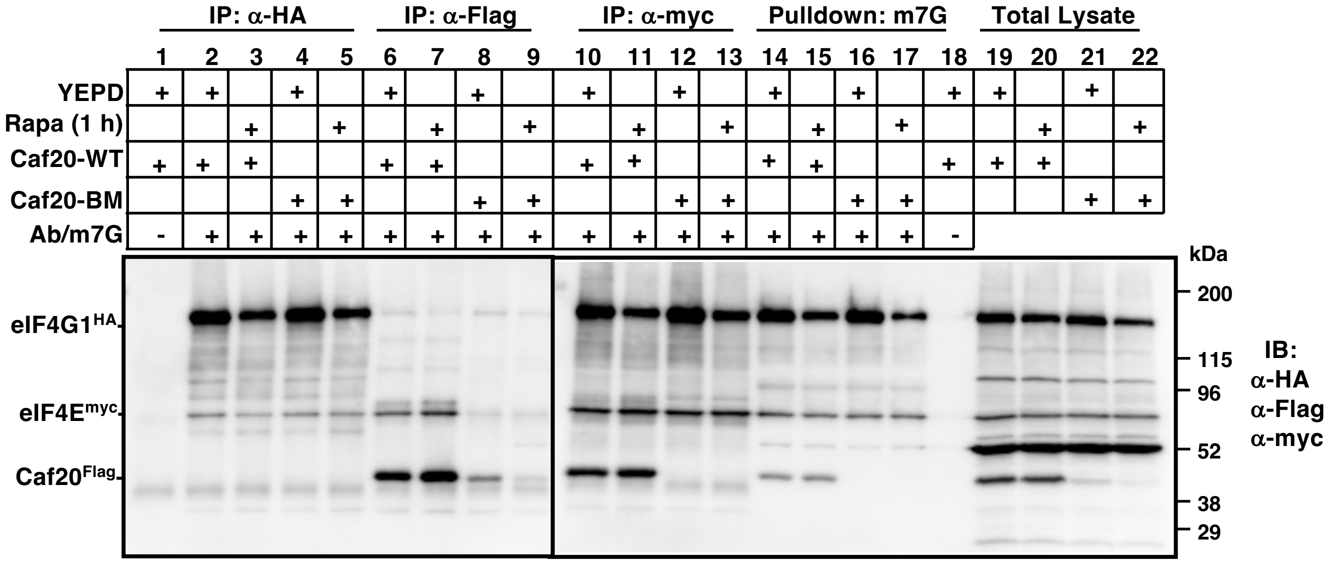

Supplement: Supplementary file 1 — DATA S1. Supporting Information. [file GTC-28-789-s001.pdf]
