# Supplementary material for: Yeast Tor complex 1 phosphorylates eIF4E‐binding protein, Caf20
Source: Genes Cells. 2023 Sep 12;28(11):789–99. doi: 10.1111/gtc.13067 (PMC11447835; doi:10.1111/gtc.13067)
Supplement: Supplementary file 2 — TABLE S1. Yeast strains used in this study. [file GTC-28-789-s002.docx]

**Table S1 Yeast strains used in this study**

*Strain Genotype Source*

BJ2168 *Mat****a*** *leu2 trp1 ura3-52 pep4-3prb1-1122 prc1-407* Laboratory stock

W303a *Mat****a*** *ade2 his3 leu2 trp1 ura3 can1* Laboratory stock

YYK1249 BJ2168 *tif4631*Δ::KanMX This study

YYK1366 BJ2168 *tif4631*Δ::KanMX *CAF20*^Flag^::HphMX *CDC33*^myc^::NatMX pRS314[TIF4631^HA^] This study

YYK1367 BJ2168 *tif4631*Δ::KanMX *caf20*Δ::HphMX *CDC33*^myc^::NatMX pRS314[TIF4631^HA^] This study

YYK1228 BJ2168 *CAF20*^HA^::KanMX *CDC33*^Flag^::HphMX This study

YYK1234 BJ2168 *EAP1*^HA^::KanMX *CDC33*^Flag^::HphMX This study

YYK1507 W303a *tor1*::NatMX::TOR1 *kog1*Δ::KanMX pRS314[^Flag^KOG1] This study

YYK1407 BJ2168 *tif4631*Δ::KanMX *CAF20-*BM^Flag^::HphMX *CDC33*^myc^::NatMX pRS314[TIF4631^HA^] This study

YYK1570 BJ2168 *tif4631*Δ::KanMX *pdr5*Δ::URA3MX pRS314[TIF4631^HA^] This study

YYK1573 YYK1366 *pdr5*Δ::URA3MX This study

YYK1575 YYK1367 *pdr5*Δ::URA3MX This study

**Table S2 Plasmids used in this study**

*Name Source*

pBluescript[3xHA] Kamada et al. (1995)

pRS314[^Flag^KOG1] Nakashima et al. (2009)

pRS314[TIF4631^HA^] This study

pET32a[CAF20] This study

**Table S3 DNA primers used in this study**

*Name Seqence*

CAF20-551new GTGCAGAAAGACCGTCGATTGAGATATCGG

CAF20-1887rcnew GTATATTTAAAGCATGAGGTAACTTTTCTGCCC

CAF20seq-952 GAGCTGTAACCTGAATATAG

CAF20-4BM CACGACATGATCAAGGCTACTATCGATGAGAGTGCACAACTGAAGCCAAG

CAF20-4BMrc CTTGGCTTCAGTTGTGCACTCTCATCGATAGTAGCCTTGATCATGTCGTG

TIF4631Xhol GCTATCCTCATCGCGACTCGAGAACGACGTGAG

TIF4631BH1rc CAGTAAGAGGATCCCCCATTAAAATGCATC

TIF46312123Not1 CTACTAAATCTGATGGCGGCCGCGCTGAAGTTGAG

TIF46312123Not1rc CTCAACTTCAGCGCGGCCGCCATCAGATTTAGTAG

EAP1-486Xhol GTGTAAAATATTTAACTCGAGATC

EAP1-3600rc GGTTCATTAATGGCAATCC

EAP1F2 GTTACCACCAGGATTAAACTCTAAAAAGAATATAAAACGGATCCCCGGGTTAATTAA

EAP1RIrc TTTGTCGTTTCCTGTCAAGTACTCGCTCGTTACGCATCGAATTCGAGCTCGTTTAAAC

CDC33-552 GTGTTCTATGTGAAAGACC

CDC33-2319rc CAACTATGTTTTGGATACC

CAF20-1001BH1 ATTTAATTTCACGACGGATCCATGATCAAGTATAC

CDC33F2 GTGCCAATGGTAGACACCCCAACCATCAATCACCTTGCGGATCCCCGGGTTAATTAA

CDC33RIrc TTAAAATAACAATTATCTTAAGAAAAATTCAGACTATCGAATTCGAGCTCGTTTAAAC

CAF20-551 GTGCAGAAAGACCGTCGATT

CAF20-1888rc CCATTTTTACTTCTATTTC

CAF20F2 CGCATTTGCTGCTTTGGAAAGTGAAGACGAAGACGACGAAGCACGGATCCCCGGGTTAATTAA

CAF20RIrc CATAGTACACGTACAGCTGCCTGAAGAAAGCATGAGCGAATGACGAATTCGAGCTCGTTTAAAC

PDR5 501 CCCTCTCTTTCCGCGGAATC

PDR5 6020rc CCAGTCGTGATCACAGTGG
